# Glycemic Control in Adult Patients With Type 2 Diabetes Mellitus Receiving Care Through a Nurse-Led Diabetic Follow-Up Clinic Versus Conventional Care: A Randomized Controlled Trial

**DOI:** 10.7759/cureus.79659

**Published:** 2025-02-25

**Authors:** Kalpana Thakur, Suresh K Sharma, Ravi Kant, Vasantha Kalyani

**Affiliations:** 1 Nursing, All India Institute of Medical Sciences, Rishikesh, Rishikesh, IND; 2 Nursing, All India Institute of Medical Sciences, Jodhpur, Jodhpur, IND; 3 Internal Medicine, All India Institute of Medical Sciences, Rishikesh, Rishikesh, IND; 4 Nursing, All India Institute of Medical Sciences, Deoghar, Deoghar, IND

**Keywords:** diabetes, glycated hemoglobin, insulin titration, nurse led clinic, treatment satisfaction

## Abstract

Background

Strengthening the role of diabetes nurse specialists, by expanding their knowledge and clinical expertise, can significantly contribute to managing diabetes mellitus and its complications.

Objectives

To assess and compare the impact of nurse-led diabetic follow-up clinics compared to conventional care on glycated hemoglobin (HbA1c) levels and diabetes treatment satisfaction among adult patients with type 2 diabetes mellitus (T2DM) seeking treatment in tertiary care settings.

Material and methods

A prospective, single-center, parallel-group randomized trial was conducted in a routine Diabetic Outpatient Department (OPD) at a tertiary care setting. After accounting for the loss to follow-up, 106 participants completed the trial. Diagnosed cases of T2DM with more than two records of HbA1c outside of the recommended range (>7%) and receiving insulin therapy for treatment were the inclusion criteria. The intervention included insulin titration and regular follow-up with the trained diabetes nurse for diabetes care and management. Endpoint measurements of HbA1c levels and diabetes treatment satisfaction were taken after three to six months of treatment to analyze the impact of the intervention.

Results

Participants receiving care through a nurse-led diabetic follow-up clinic had a decrease in HbA1c levels after the intervention, with an average paired difference of 1.75 (1.12 to 2.38), statistically significant at the 0.05 level of significance. Although HbA1c levels in the Control Group also decreased after the standard intervention, with a mean paired difference of 0.64 (0.03 to 1.25), it did not reach statistical significance, with a p-value of 0.05 at the 0.05 level of significance. However, patients' HbA1c levels between the Intervention and Control Groups at baseline (p = 0.101) and post-intervention (p = 0.369) were not statistically significant at the 0.05 level of significance. Furthermore, participants in the Intervention Group achieved better glycemic control, as measured by self-reported satisfaction with regards to hyperglycemia (-0.95 ± 1.43 vs. 0.39 ± 1.88, p = 0.001) and hypoglycemia (-0.35 ± 1.02 vs. 0.71 ± 1.1, p < 0.001) levels.

Conclusion

The nurse-led diabetes follow-up clinic was found to be an invaluable service, and the addition of a diabetes specialist nurse as part of the treatment team can alter the conventional pathway for diabetes treatment.

## Introduction

Type 2 diabetes mellitus (T2DM) is a heterogeneous metabolic condition characterized by hyperglycemia, insulin resistance, and impaired insulin secretion. Furthermore, prevalence rates estimate that diabetic cases are rising more quickly in low- or middle-income countries than in high-income nations (36.78 crores vs. 9.52 crores, respectively). This finding was supported by the global burden of disease research, which noted that an increasing population and older adult demographic can account for increased diabetic incidence rates in large population nations like India and China [1,2]. According to the International Diabetes Federation (IDF) Atlas Factsheet 2021, diabetes in India has reached epidemic proportions, with 90 million diagnosed cases, or 1 out of 11 adults suffering from diabetes, and accounting for 7,47,000 deaths annually. Without timely action being taken on the prevention of diabetes and related complications, its prevalence could mount by 69%, further rising to 152 million by 2045 [3].

Maintaining optimal blood glucose levels in patients living with diabetes mellitus is both challenging and essential to minimizing the risks of microvascular and macrovascular complications, while simultaneously improving the mental well-being and quality of life of individuals living with the condition. Diabetes is a complex medical condition; therefore, standard treatments may not always meet each patient's individual and unique needs [4,5]. Personalized medicine has emerged as a cornerstone in diabetes management, offering improved patient outcomes and providing more cost-effective and patient-centric care worldwide. New findings in research indicate that lifestyle and pharmaceutical interventions could provide effective ways of controlling hyperglycemia and avoiding complications [6,7].

The latest models for diabetes mellitus care in the West indicate that diabetes nurse specialists can independently manage nurse-led clinics to enhance glycemic control and improve patient satisfaction [8-11]. South East Asian countries, on the other hand, still do not accept nursing professionals as skilled professionals who can effectively run nurse-led clinics. Additionally, there are no prescription rights available for nurses, which further restricts the role of nurses when it comes to patient treatment [12-14]. According to the recent World Health Organization (WHO) reports, India may have achieved the recommended doctor-patient ratio of 1:1000 but lacks specialized doctors. For example, there are only 650 diabetes specialists available in India, which could be a reason for increased diabetic complications [15].

It is important to understand that nurse-led insulin titration and prescription should always be done within a clearly defined framework and approved standard guidelines. Although, in the Indian scenario, nurses are not permitted to prescribe medication, if trained and specialized nurses manage it, they may be given the authority to titrate dosages of insulin that will improve the patient's glycemic control. Numerous studies have been conducted on nurse-led interventions for diabetes management, and all have focused on exploring the effectiveness of various educational interventions in managing T2DM; however, none has explored in detail how nurses may play an active role in insulin titration and the management of T2DM. Therefore, this study aims to assess and compare the difference in glycated hemoglobin (HbA1c) levels and treatment satisfaction of adult patients with T2DM receiving care through nurse-led diabetic follow-up clinics vs. conventional care before and after the intervention.

## Materials and methods

The present randomized controlled trial is a prospective, single-centric, parallel-group study with block randomization. The study is best described as a pragmatic randomized controlled trial because it was carried out in a routine Diabetic Outpatient Department (OPD) at All India Institute of Medical Sciences, Rishikesh, India, during the year 2018-2019 to compare the effectiveness of a nurse-led diabetic follow-up and conventional care in general practice for the management of T2DM patients, considering the important principles of randomization, control, and manipulation, which can produce reliable and valid evidence for clinical practice. Computer-based two-block randomization was used, and the allocation was concealed by using sequentially numbered opaque sealed envelopes with the SNOSE (Sealed, Numbered, Opaque, Sequential Envelopes) technique. The envelope was opened by a staff nurse posted in the Medicine OPD, who had not been involved in the care of the recruited patients. Non-interventionists, including laboratory technicians, assessed the study outcomes by evaluating HbA1c reports, and statisticians who analyzed research data were blinded to the treatment allocation to minimize assessor bias.

The main study was carried out from March 2020 to January 2022 using purposive sampling techniques to recruit subjects seeking treatment for T2DM at the Diabetic OPD clinic. Patients diagnosed with T2DM within the age group of 18 years with more than two records of HbA1c outside of the recommended range >7% and receiving insulin therapy for treatment were included in the study. All the participants who were eligible and willing to participate in the study signed the written informed consent form before participating. Patients with gestational diabetes, serious or life-threatening illnesses, and diabetic patients who were not able to communicate or do follow-up regularly over the phone were excluded. The trial was registered in CTRI with reference number REF/2018/12/023061. It was also approved by Institutional Ethical Committee-wide letter no. AIIMS/IEC/19/831. Calculation of sample size was performed on the basis of change in HbA1c levels found in earlier trials using the formula [11,16]. \[ N = \frac{2 \sigma^2 \left( Z_{1-\alpha/2} + Z_{1-\beta} \right)^2}{(\mu_1 - \mu_2)^2} \]

In order to detect a difference of this magnitude that is significant with 95% confidence and a power of 80%, including a 20% attrition rate, the calculated sample size was 120, i.e., 60 patients in each group. After accounting for loss to follow-up in the present study, a total of 106 T2DM patients completed the trial: 51 in the Control Group and 55 in the Intervention Group.

The nursing intervention included coordinated care between the nurse, physician, and diabetic patient. The intervention was performed by a nurse researcher, who was also a certified and trained diabetic educator. The process of carrying out the intervention, specifically the titration of insulin dosage, was based on written and signed instructions from the physician and ethics committee of the institute. The content used by nurse researchers for education, demonstration, and skill training purposes was developed after a review of guidelines from the IDF and WHO. The nurse researchers met the regulatory reporting requirements for unexpected serious adverse drug effects by reporting them to regulatory authorities. The nurse researcher, who was the interventionist in the study, conducted a 15-minute initial screening session for patients to conduct pre-test assessments. The patient attended a detailed face-to-face session in the nurse-led diabetic follow-up clinic for 30 minutes, where education on diabetes and its management was given, along with demonstrations and skill training on Self-Monitoring of Blood Glucose (SMBG), the seven-point blood glucose profile, and administration of insulin. The patient was also educated on reporting and managing hypoglycemic emergencies at home. At each follow-up phase of the intervention, a nurse researcher made direct calls to study subjects for 15 minutes twice weekly throughout their follow-up phase and performed dosage titration or adjustment of insulin based on written directives or orders issued telephonically by physicians, if required. The nurse researcher also addressed psychological and motivational barriers and provided guidance in case of any doubts by sharing recorded video lectures virtually to bolster education and training provided during initial discussions. The Control Group patients received routine or standard nursing care in the Diabetic OPD Clinic. The care was provided by doctors and registered nurses at the diabetic clinic. There was no follow-up or insulin titration by a nurse. The supplies of Accu-Chek glucometers, lancets, glucometer strips, and insulin were purchased from Roche Diabetes Care India Private Limited, Mumbai, India, and were distributed equally to both groups (Intervention Group and Control Group). This was done to avoid discrepancies in the services provided by the institute and to ensure that there would be no bias in the results of the research.

The present study measured the HbA1c levels and the diabetes treatment satisfaction of patients at baseline and within three to six months after the intervention. Recruitment for this study took over two years due to disruptions in participant entry caused by a few restrictions associated with the COVID-19 second wave. The study followed a set schedule for follow-up; patients were monitored from three to six months following their initial visit. The nurse researcher continued to run nurse-led follow-up diabetic clinics to eliminate discrepancies and differences in intervention methods. The baseline proforma included information on the sociodemographic and clinical profiles of the patients, gathering baseline information about patients diagnosed with T2DM. A recording sheet was used to compare pre- and post-intervention HbA1c levels, as evaluated by lab investigations. DTSQ and DTSQc were the standardized tools used to assess the change in diabetes treatment satisfaction over the treatment duration among the patients in both groups [17]. At the conclusion of the trial, post-test assessments of all the outcomes were evaluated for all study participants in both the Intervention and Control Groups.

The data analysis was planned carefully, and IBM SPSS Statistics for Windows, Version 26 (Released 2019; IBM Corp., Armonk, NY, USA), was used to analyze the data. Descriptive and inferential statistical methods were planned according to the objectives and hypotheses of the study. Descriptive statistics were used to describe sample characteristics, while inferential statistics were used to measure the difference between outcome variables within and between groups. The primary method of analysis for this study was the intention-to-treat analysis.

## Results

The trial was completed by 106 participants, 55 of whom were in the Intervention Group and 51 in the Control Group. The mean age (years) of participants in the Intervention Group was 52.11, and in the Control Group, it was 51.82. In the Intervention Group, around 33 (60%) participants were men, while the Control Group had 28 (54.9%) men. Participants in the Control Group had a duration of 9.41 years since they were diagnosed with diabetes, compared to 9.86 years among participants in the Intervention Group. Patients in both groups suffered from comorbidities in almost equal proportions. Around 22 (40.0%) participants in the Intervention Group had been taking insulin for the last two to five years, while in the Control Group, 25 (49%) participants were insulin users with a history of two to five years. It was clearly evident that there was no statistically significant difference between the two groups in terms of their baseline characteristics, with a p-value of >0.05 (Table 1).

**Table 1:** Baseline characteristics of study participants (N = 106) Note: No statistically significant difference was observed between the groups, with all p-values >0.05.

Characteristics	Intervention group (n = 55)	Control group (n = 51)	p-value
Age (in years), mean ± SD	52.11 ± 13.41	51.82 ± 10.89	0.9
Gender			0.59
Female	22 (40.0%)	23 (45.1%)	
Male	33 (60.0%)	28 (54.9%)	
Place of residence			0.79
Urban	39 (70.9%)	35 (68.6%)	
Rural	16 (29.1%)	16 (31.4%)	
Type of family			0.9
Joint	20 (36.4%)	18 (35.3%)	
Nuclear	35 (63.6%)	33 (64.7%)	
Extended	00 (0.0%)	00 (0.0%)	
Educational status			0.67
Non-formal	14 (25.5%)	09 (17.6%)	
Primary	04 (7.3%)	07 (13.7%)	
Higher secondary	23 (41.8%)	22 (43.1%)	
Graduate and above	05 (9.1%)	03 (5.9%)	
Professional	09 (16.4%)	10 (19.6%)	
Occupation			0.52
Unemployed	00 (0.0%)	00 (0.0%)	
Unskilled worker	16 (29.1%)	10 (19.6%)	
Skilled worker/professional	19 (34.5%)	20 (39.2%)	
Housewife	20 (36.4%)	21 (41.2%)	
Duration since diagnosed with diabetes type II (in years), mean ± SD	9.86 ± 6.19	9.41 ± 5.61	0.44
Co-morbidity status			0.3
No	13 (23.6%)	08 (15.7%)	
Yes	42 (76.4%)	43 (84.3%)	
Duration of receiving insulin therapy			0.64
≤1 year	28 (50.9%)	22 (43.1%)	
2 to 5 years	22 (40.0%)	25 (49.0%)	
>5 years	05 (9.1%)	04 (7.8%)	

Wilcoxon signed-rank tests were employed to examine differences within groups, while Mann-Whitney U tests were utilized to analyze any disparities between them. Researchers observed that participants in the Intervention Group found their HbA1c levels decreased after six months of nursing intervention by an average paired difference of -1.75 (-1.12 to -2.38); this difference was statistically significant at the 0.05 level of significance. Although HbA1c levels in the Control Group also decreased after the standard intervention, with a mean paired difference of -0.64 (-0.03 to -1.25), it did not reach statistical significance, with a p-value of 0.051 at the 0.05 level of significance. Changes in patients' HbA1c levels between the Intervention Group and Control Group at baseline (p = 0.101) and post-intervention (p = 0.369) were not statistically significant at the 0.05 level of significance (Table 2).

**Table 2:** Comparison of HbA1c levels of all the participants during study period (N = 106) Note: *Statistically significant difference observed at p-values <0.05.

Clinical outcomes variables	Intervention group (n = 55)		p-value difference within group
	Baseline	Mean paired difference after 6 months (95% CI)	
HbA1c level	10.62±1.757	-1.75 (-1.12, -2.38)	0.00*
	Control group (n = 51)		
	10.10 ± 2.077	-0.64 (-0.03, -1.25)	0.05
p-value difference between the group	0.101	0.369	-

Mann-Whitney U test was used to compare all components of treatment satisfaction and glycemic management scores between both groups after the intervention. At a 0.05 level of significance, statistically significant differences were identified between the Intervention and Control Groups for convenience, flexibility, understanding, and continuity of treatment. Furthermore, participants in the Intervention Group achieved better glycemic control, as measured by self-reported satisfaction with regards to hyperglycemia (-0.95 ± 1.43 vs. 0.39 ± 1.88, p = 0.001) and hypoglycemia (-0.35 ± 1.02 vs. 0.71 ± 1.1, p = <0.001) levels (Table 3).

**Table 3:** Mean change scores of diabetes treatment satisfaction between the Intervention and Control Group participants (N = 106) Note: *Statistically significant difference observed between groups at p-values <0.05. The data are expressed as mean ± SD, mean difference, and median (Min to Max) unless otherwise stated. DTSQ, Diabetes Treatment Satisfaction Questionnaire

Domains	Components of DTSQs and DTSQc	DTSQs Diff scores baseline mean ± SD median (Min to Max)		p-value difference between groups	DTSQc scores after 6 months mean ± SD median (Min to Max)		p-value difference between groups
		Intervention (n = 55)	Control (n = 51)		Intervention (n = 55)	Control (n = 51)	
								Treatment satisfaction	Satisfaction	-0.81 ± 0.77	-1.07 ± 0.75	0.103	1.45 ± 1.18	1.04 ± 1.34	0.102
-0.50 (-2.00, 0.50)	-1.00 (-3.00, 0.50)	2.00 (0.00, 3.00)	1.00 (-2.00, 4.00)												
Convenient	-0.77 ± 0.79	-1.24 ± 0.68	0.002*	1.36 ± 1.12	0.41 ± 1.15	<0.001*		
	-0.50 (-2.00, 0.50)	-1.00 (-2.50, 0.50)		2.00 (0.00, 3.00)	0.00 (-2.00, 2.00)			
Flexible	-0.92 ± 0.74	-1.20 ± 0.63	0.044*	1.29 ± 1.13	0.35 ± 1.03	<0.001*		
	-1.00 (-2.50, 0.50)	-1.50 (-2.50, 0.00)		2.00 (0.00, 3.00)	0.00 (-2.00, 2.00)			
Understanding	-0.77 ± 0.84	-0.78 ± 0.75	0.859	1.36 ± 1.00	0.92 ± 1.03	0.032*		
	-0.50 (-2.50, 0.50)	-0.50 (-2.50, 0.50)		2.00 (0.00, 3.00)	1.00 (-2.00, 3.00)			
Recommend	-1.21 ± 0.93	-1.32 ± 0.61	0.709	1.27 ± 1.39	0.76 ± 0.95	0.071		
	-1.00 (-2.50, 0.50)	-1.50 (-2.50, 0.00)		0.00 (-1.00, 3.00)	0.00 (-1.00, 3.00)			
Continue	-1.08 ± 0.88	-1.22 ± 0.72	0.324	1.42 ± 1.39	0.90 ± 1.15	0.034*		
	-1.00 (-3.00, 0.00)	-1.50 (-2.50, 0.50)		2.00 (0.00, 3.00)	1.00 (-1.00, 3.00)			
Glycemic control	Hyperglycemia	-2.83 ± 0.99	-2.13 ± 0.91	<0.001*	-0.95 ± 1.43	0.39 ± 1.88	<0.001*
		-3.00 (-4.00, 0.00)	-2.50 (-4.00, -0.50)		-1.00 (-3.00, 3.00)	0.00 (-2.00, 4.00)	
	Hypoglycemia	-1.10 ± 1.16	-0.64 ± 0.78	0.063	-0.35 ± 1.02	0.71 ± 1.17	<0.001*
		-0.50 (-4.00, 0.00)	-0.50 (-2.50, 1.00)		0.00 (-3.00, 3.00)	1.00 (-3.00, 2.00)	

The Kruskal-Wallis and Mann-Whitney U tests were used to find the association of clinical outcomes, i.e., HbA1c levels and diabetes treatment satisfaction, with participants’ demographic and clinical characteristics at the 0.05 level of significance. Participants in the Intervention Group had a significant association between satisfaction and glycemic control with gender, at a p-value = 0.032. Similarly, patients in the Control Group were found to have a significant association between treatment satisfaction and gender, at p = 0.013. Participants’ HbA1c levels had no significant association with any of the demographic and clinical variables at the 0.05 level of significance (Table 4).

**Table 4:** Association details of the clinical outcomes of study participants with demographic variables in the Intervention Group (N = 106) Note: *Statistically significant difference observed at p-values <0.05.

Demographic characteristics	Intervention group (n = 55)						Control group (n = 51)					
	HbA1c level		Satisfaction with treatment		Satisfaction with glycemic control		HbA1c level		Satisfaction with treatment		Satisfaction with glycemic control	
	H/Z value	p-value	H/Z value	p-value	H/Z value	p-value	H/Z value	p-value	H/Z value	p-value	H/Z value	p-value
Age	0.49	0.78	0.06	0.97	0.27	0.88	4.02	0.13	1.76	0.42	1.37	0.51
													Gender	0.79	0.43	-0.38	0.70	2.15	0.03*	-1.44	0.15	2.49	0.013*	-0.57	0.57
Place of residence	1.90	0.06	-0.72	0.47	0.83	0.41	0.62	0.53	-1.43	0.15	0.65	0.52													
													Type of family	0.75	0.39	2.37	0.12	2.76	0.10	0.00	0.96	1.04	0.31	0.13	0.72
Educational status	1.28	0.86	3.10	0.54	3.23	0.52	3.42	0.49	8.40	0.08	7.41	0.12													
													Occupation	0.50	0.78	1.90	0.39	4.87	0.09	4.09	0.13	4.48	0.11	0.99	0.61
Duration since diagnosed with diabetes type II	0.65	0.72	4.28	0.12	0.05	0.97	1.48	0.48	0.45	0.80	0.41	0.81													
Co-morbidity status	0.66	0.51	0.80	0.43	0.23	0.82	-0.72	0.49	2.16	0.03*	0.84	0.40
													Duration of receiving insulin therapy	0.03	0.99	2.34	0.31	1.08	0.58	2.73	0.26	0.19	0.91	2.08	0.35

## Discussion

Diabetes and its complications cause morbidity and even mortality among patients, and despite advances in treatment, there remains no one-size-fits-all solution. Individualized care must be tailored specifically to each patient based on individual needs; unfortunately, due to a limited number of endocrinologists, this may become challenging [4,5]. This trial marks the first randomized controlled trial conducted in Indian settings, where nurse-led insulin titration was undertaken for patients attending nurse-led diabetic follow-up clinics run by trained diabetic nurses. Nurse-led diabetes follow-up proved an effective means of achieving better glycemic control and treatment satisfaction than receiving standard treatment options.

Our results were in line with previous studies [8,9,11], which found that there was a significant improvement in glycemic control for patients who received treatment from a nurse. However, the Standard Care Group did not show any improvement. In contrast, a study conducted in the Netherlands found no significant differences in HbA1c target levels between standard treatment and nursing intervention [10]. On the contrary, a similar study to ours, focusing on the effects of assigning a nurse-care manager who was permitted to titrate medications for glycemic profiles, blood pressure, and lipid profiles following specific protocols, showed that the Intervention Group also had significant reductions in HbA1c, total cholesterol, and blood pressure at one year, when compared with the Usual Care Group [18].

Treating chronic illnesses such as diabetes mellitus requires high-quality care, and treatment satisfaction, measured through patient-reported outcomes, is an integral component of that quality. According to these research studies, nurse-led diabetic follow-up had a positive influence on patients' treatment satisfaction scores. Participants enrolled in the nurse-led diabetes Intervention Group reported being more motivated, receiving greater support, and being better at self-management when facing physical illness or emotional stress [19,20]. It was also discovered in related research that treatment satisfaction was strongly correlated with BMI and other factors, including medical conditions and medications, hyperglycemia perception frequency, SMBG therapy, lifestyle modification strategies, comorbidities, or complications [21]. On the contrary, this study revealed that gender and comorbidity factors of Control Group participants had a statistically significant relationship with treatment satisfaction, while nurse-led diabetes Intervention Groups were not associated with any clinical or demographic variables.

Gaining better glucose control reduces morbidity and extends life expectancy while simultaneously improving the quality of life for patients. According to this study's results, participants receiving standard diabetes treatment experienced an increase in perceived hyperglycemia and hypoglycemia, which is consistent with literature that concluded that nurse-led Intervention Group participants reported hypoglycemia episodes more often than Standard Care Group participants in terms of accurate documentation. When compared to conventional diabetes care and treatment, nurse-led diabetic follow-up clinics were more effective in terms of glycemic control and patient treatment satisfaction. This approach acts as a valuable addition to diabetes management, where nurses can serve as diabetes specialists who offer customized advice to their patients as they receive treatments to control diabetes and prevent complications.

Study limitations

Nurse-led clinics for managing diabetes can have profoundly positive ramifications for patient care, healthcare systems, and nursing practice. These clinics can be a great way to expand access to healthcare, particularly in rural or underserved areas. The expertise of nurses allows them to fill the void, ensuring patients receive timely and appropriate care while preventing long-term complications. This article is one part of a larger study, and the additional findings will be presented in subsequent publications. As this study focused solely on one tertiary care setting and was conducted over just three to six months, the results cannot be generalized to changes over a longer timeframe. Based on the findings of this study, evidence-based guidelines and protocols for diabetic patient care can be implemented in clinical settings to enhance the quality of treatment and outcomes. Further studies may explore the long-term impacts of nurse-led diabetic follow-up interventions on controlling patients' glycemic levels, as well as the cost-effectiveness of such clinics within healthcare systems.

## Conclusions

The present study shows that the HbA1c levels have improved after nursing intervention. The standard treatment resulted in a perceived increase in hyperglycemia or hypoglycemia in the Control Group, which suggests a poorer outcome. The Intervention Group patients reported that regular contact and follow-ups with a caring and experienced diabetic nurse researcher were the keys to improving their glycemic control. Participants who attended nurse-led follow-ups were more satisfied with their diabetes treatment. Nurse-led follow-up diabetes clinics can improve the HbA1c level and increase treatment satisfaction for T2DM patients who are poorly managed. This was an excellent adjunct to standard medical care for these patients, who required more specialist attention.
