# Correction: Glycemic Control in Adult Patients With Type 2 Diabetes Mellitus Receiving Care Through a Nurse-Led Diabetic Follow-Up Clinic Versus Conventional Care: A Randomized Controlled Trial

**DOI:** 10.7759/cureus.c215

**Published:** 2025-03-31

**Authors:** Kalpana Thakur, Suresh K Sharma, Ravi Kant, Vasantha Kalyani

**Affiliations:** 1 Nursing, All India Institute of Medical Sciences, Rishikesh, Rishikesh, IND; 2 Nursing, All India Institute of Medical Sciences, Jodhpur, Jodhpur, IND; 3 Internal Medicine, All India Institute of Medical Sciences, Rishikesh, Rishikesh, IND; 4 Nursing, All India Institute of Medical Sciences, Deoghar, Deoghar, IND

This article has been corrected as follows. The appendices of this article previously included the Diabetes Treatment Satisfaction Questionnaire and Diabetes Treatment Satisfaction Questionnaire. These questionnaires were included without the necessary permissions from Health Psychology Research Ltd. They have now been removed from the article. This correction does not impact the study’s results, analysis, or conclusions.

